# Case Department

**Published:** 1889-01

**Authors:** 


### CASE DEPARTMENT.

FRACTURE OS SUFFRAGINIS.—During the winter of 1887–88, I was called to see a mare, extremely lame and suffering from an injury of hind leg. I found a fracture of os suffraginis. The mare was a valuable trotting animal; in foal to a standard bred stallion, seven months pregnant, and quite heavy. I was of the opinion that treatment could not be pursued successfully unless the animal was put in slings, the owner being desirous of the well-being of the progeny. Such a case had never presented itself to me before, and I was quite anxious to know if any such experience had been chronicled, but could find none in any book of reference on which I could place my hand. I arranged the fracture merely with wire and ash splints from the shoe up, and put the mare in slings, keeping her there three months. She foaled a month afterwards all right, the foal thriving well. When the foal was four months old, an enlargement appeared on the pastern similar to the callus on the pastern of the mare, of a firm consistency, but is fast disappearing without treatment. This mare has since fractured shaft of ileum on same side, is in slings now, probably a subject of fragilitis ossium, and bids fair to recover. I was formerly of the opinion that placing the mare in slings would cause abortion, or at least a malformation or mal-position. After being released from them she would invariably lie down on injured side and could not get up, had to be turned over on sound side. Not the slightest ill-effects were observed.

AMPUTATION OF UTERUS IN MARE.—During summer of 1887, was called hurriedly to see a mare reported bleeding to death. I found that the animal foaled about two hours before. A person was sent for who was supposed to *know about all such things* and mistaking the uterus for afterbirth, commenced to vigorously pull at it until he made a large rent in it. There was profuse hemorrhage, the animal down and partly amaurotic. I told the owner the mare would die and prepared to go away. An extravagant fee was offered if I would do something, and I decided to try amputation, although I had operated many times, always with unsuccessful results both in mares and cows. Assisted by two strong carmen, I applied a strong quadruple ligature close up to the neck, and removed the body with one sweep of the knife. The ends of the ligatures flew back in the vagina out of sight, as if there had been a spring at the end. I cut the ends too short, but I did not care much whether they were too short or too long; ordered a stimulant and comfortable quarters. Next day the animal staggered to its feet only to fall headlong, and bruised itself considerably. The pulse being moderately strong somewhat surprised me; a warm drink containing stimulant, was taken voluntarily during the night. The cries of the foal aroused the animal to her feet, where she remained. Urine was removed with a catheter thrice daily, and mare received every attention. Quantities of a sanguineous fluid was ejected per virgnium. On fourth day the animal becoming restless for foal, it was put alongside, On fifth day shreds of pus were ejected. On the sixth day large flakes passed frequently, the animal attempting to urinate every little while, quantities of pus coming away at each attempt. On eighth day ligatures came away, and violent symtoms of colic made their appearance. It took me some time to find out that this was caused by constipation of bowels, which was quickly removed by enemas. The improvement from this out was rapid. The mare and foal did well, the former now working about the streets, apparently none the worse. I do not take any particular credit for this operation, nor was it done in a very scientific manner, but entirely an exceptional case which cannot be accounted for.

Abscess of Liver, (Calf).—During the past summer I was asked to hold a post-mortem on bull-calf, six months old, which had died suddenly during the night without any premonitory symptoms. This animal was a highly bred Holstein, and was fed heavily on everything it would eat, with the object of forcing it along so that it might serve cows at an early age. Nothing appeared amiss the day before, and outwardly it would be said to be in the pink of condition. On opening the abdominal cavity a quantity of clear laudable pus showed itself on the abdominal walls, which was quickly traced to the liver, and the whole gland was a regular network of smaller abscesses, varying in size from a hazelnut to a walnut. It could hardly be conceived that an animal in this condition would appear so unusually healthy, but it was so; the coat shone like an otter. The cause I have no doubt was due to injudicious feeding, causing repeated attacks of congestion, and resulting abscesses. I do not think the liver, at least in young animals, to be a very sensitive organ. I have repeatedly, when spaying young bitches, nipped a piece off the liver with my finger nail or forceps, without causing the slightest ill-effects, more than it would bleed rather freely, as the escape of a moderate quantity of blood into the abdominal cavity of young bitches, does no harm.

*St. John, N. B.* JAMES H. FRINK,
*Government Veterinary Inspector.*

---

ON THE OPERATION FOR PREPARING A SKUNK (MEPHITIS) FOR A ZOÖLOGICAL GARDEN.—This operation, as the reader no doubt has already anticipated, from reading the above title, consists in removing the anal glands from the living animal, and thus deprive him of the power of ejecting that nauseous fluid which renders him so offensive during life, and for which he is nearly universally dreaded and shunned.

Having captured your specimen alive and unhurt in an ordinary "box-trap," without irritating him he must be carefully transferred to a rather smaller box with sliding cover, which tightly fits it. Then take a sponge as large as an ordinary orange, tie it with a string, and pour upon it about an ounce of Sulphuric æther, lowering it down into the box in front of him, and closing the cover. If he shifts his position you can easily follow him about with the sponge by means of the string tied to it. These animals, so far as my observation goes, seem to be quite susceptible to the influence of this ænesthetic, and usually succumb to it in about ten, or at the most fifteen minutes, when applied as here directed, the box being kept carefully closed.

He can now be removed from the latter, *lifting him by the tail*, and placed upon a small deal table in a good light for the operation. For this the following instruments are required, viz:—two well-waxed silk ligatures; a tenaculum; a small pair of sharp, strong scissors, *curved on the flat*; a small probe; and finally, a few clean dressing sponges, a basin of cold water, and the usual appliances to maintain the ænesthetic condition in the animal, for this operation should take half an hour at least to perform.

Placing the animal on his back, direct one of those assisting you to attend to the administration of the æther; while another holds the hind limbs of the animal for you in such a manner as not to have them in the way. A search with the probe soon reveals the position of the *two* glands which secrete the offensive fluid, of this otherwise exceedingly interesting little mammal. They are well within the margin of the anal orifice, one on either side, and opposite each other transversely, being hidden in each case in a sulcus, or fold of the mucous membrane, though easily discoverable through the papilla which forms the neck and opening of either gland. Each of these papilla are several millimetres long, nearly cylindrical in form, rounded on top, where also is to be seen the minute orifice constituting the external opening for the exit of the gland's secretion. Carefully hook your tenaculum into one of the papilla, and by gentle traction lift it from its hiding place; then direct some one to hold this instrument, while you pass one of the ligatures around the papilla, and firmly ligate it. Next perform the same operation upon the remaining papilla of the opposite side, and you are then safe so far as any danger is concerned as to the escape of the contents of the glands, and in the vast majority of cases there is no reason why an accident of this kind should have previously occurred.

Taking one of the ligatures in your left hand put it gently on the stretch, pulling the papilla out beyond the orifice of the anus, and placing the surrounding tissues in a proper degree of tension. With the curved scissors in your right hand, carefully nick through the mucous membrane all around the base of the papilla; and then commence dissecting out the gland with the same instrument, keeping the convex surfaces of the blades *next* the gland, and proceeding by careful clips upon its surface, separating the organ from the surrounding tissue in which it is seen to be embedded. All this time the ligature in your left hand must be kept firmly on the stretch, and great care exercised that you do not cut into the rectum on the one hand, nor into the gland on the other. The first might retard the healing process, while the results of the latter can perhaps be better imagined than they can be awarded space to vividly portray them here. We should allot at least ten or twelve minutes to the complete removal of one of these organs, which can be nicely accomplished in that time, when the wound should be carefully sponged out, and the opposite gland immediately removed in precisely the same manner.

Sponging with cold water should now follow until all hemorrhage ceases, and all signs of blood completely removed from the fur, the parts nicely cleaned, and then allow your *Mephitis* to come out from his sleep. Recovery is usually rapid and complete, the wound healing up in the most satisfactory manner. Upon removing these glands from a full-grown animal, each are seen to be about as large as a good-sized almond, somewhat pear-shaped, although rather compressed from side to side. I have never made personally, a histological investigation of one, but valuable contributions to this part of the subject have been made by Wyman (*Pr.* Bost. Soc., i. p. 110, 1844), Warren (*loc. cit.* iii, p. 175, 1851), Parker (*Ann. Nat*., v, p. 246, 1871), Chatin (*Ann. Sci. Nat.*, [5] xix, p. 100, 1874), and others. If we plunge a sharp instrument into one of these glands after they have been removed, the fluid seems to nearly all escape in the first gust that follows, leaving the organ quite placid, and apparently empty.

When in contact with the skin it is more or less irritating in its effects, smarting like mustard does when mixed with a dilute acid, and freshly applied. There is another thing in regard to this fluid which I have failed to find noted elsewhere, and that is this, —if a little is spread out on a non-absorbing surface, and the flies gather about, and upon it you will soon see a number of them tumble over in a stupified condition, just as though they had imbibed some toxic fluid, and they do not seem to recover again, either, but remain quite lifeless, and apparently dead.

Skunks thus robbed of their sole objectionable feature, prove themselves to be among the most engaging of all our smaller mammals, and make wonderfully interesting and instructive pets.

R. W. SHUFELDT, M. D.

*Fort Wingate, New Mexico.*

---

UPWARD DISPLACEMENT OF THE EYE IN THE HORSE.—During the summer of 1886, I was requested by the owner of a neighboring cottage on the New Hampshire coast to look at one of his carriage horses which had met with a curious accident. The groom, on going to the stall in the morning, found the horse lying down with the halter jammed tight around the top of the head. The large iron ring of the halter was forced strongly into the socket of the right eye edge-wise. Removing the halter, to his surprise, the orbit was empty save for the presence of a large blood clot. Immediately above the edge of the orbit, however, in place of the fossa which should exist at that point, there was quite a prominent tumor. By gentle manipulation he succeeded in reducing this swelling, and the eye ball slipped back into its proper place. It had been forced up through the foramen which exists in the roof of the orbit until it was entirely outside of the cavity. I saw it three days after the accident. There was then considerable extravasation under the conjunctiva with some conjunctivitis, and and a moderate purulent discharge. The eye ball was slightly protuberant. On depressing the lower lid I could distinctly see the torn surface of rectus inferior muscle close to its attachment to the ball. This healed kindly and the movements of the eye became quite normal. I have not learned whether there was impairment of vision as a permanent result, but from an examination of the orbit in the skeleton, I am led to think that the change of position described might take place without rupturing or even excessively stretching the optic nerve, and that such a result was therefore not necessarily to be looked for.

BENJAMIN LEE, A. M., M. D., Ph. D.,
*Secretary of the State Board of Health of Pennsylvania.*
*1532 Pine St., Philadelphia.*

